# Predictive and Experimental Approaches for Elucidating Protein–Protein Interactions and Quaternary Structures

**DOI:** 10.3390/ijms18122623

**Published:** 2017-12-05

**Authors:** John Oliver Nealon, Limcy Seby Philomina, Liam James McGuffin

**Affiliations:** School of Biological Sciences, University of Reading, Reading RG6 6AS, UK; j.o.nealon@pgr.reading.ac.uk (J.O.N.); l.s.philomina@pgr.reading.ac.uk (L.S.P.)

**Keywords:** protein, interaction, prediction, homology, docking, quality assessment, experimental

## Abstract

The elucidation of protein–protein interactions is vital for determining the function and action of quaternary protein structures. Here, we discuss the difficulty and importance of establishing protein quaternary structure and review in vitro and in silico methods for doing so. Determining the interacting partner proteins of predicted protein structures is very time-consuming when using in vitro methods, this can be somewhat alleviated by use of predictive methods. However, developing reliably accurate predictive tools has proved to be difficult. We review the current state of the art in predictive protein interaction software and discuss the problem of scoring and therefore ranking predictions. Current community-based predictive exercises are discussed in relation to the growth of protein interaction prediction as an area within these exercises. We suggest a fusion of experimental and predictive methods that make use of sparse experimental data to determine higher resolution predicted protein interactions as being necessary to drive forward development.

## 1. Introduction

Accurately predicting how a protein folds, functions and interacts with other molecules, remains one of the most critical problems in bioinformatics. An entirely algorithmic solution to elucidate protein folding would be ideal, and a folding process of some form must exist in nature as postulated in Levinthal’s paradox [1]. However, discovering such a solution has eluded the modelling community and as a result, the most effective methods of protein fold prediction use template based approaches. Existing protein structures are compared to the sequence that is being predicted utilizing the extensive library of protein structures and complexes assembled from experimental procedures such as electron microscopy, nuclear magnetic resonance (NMR) spectroscopy, X-ray crystallography and small-angle X-ray scattering (SAXS). X-ray crystallography gives a complete, high-resolution analysis of the three-dimensional structure of proteins, but large, well-ordered single crystals are required. Electron diffraction and electron microscopy (EM) are used mainly where crystals cannot be obtained due to very large complexes, but the drawback of these methods is low-resolution structures. Even though NMR is the leading method for structure determination, after X-ray crystallography, it has limitations in terms of sensitivity, selectivity and target analysis [2,3]. SAXS can be carried out using a wide variety of sample conditions from frozen to the natural solution, although it is lower resolution than NMR and X-ray crystallography [4].

Determining protein structures and their function from sequence data is an essential part of modern molecular biology and leads onto the problem of determining the protein interacting partners and quaternary structural state. A thorough review of scientific literature and progressively reliable computational predictions have resulted in the formation of massive databases of protein interaction data. These databases have become an essential resource for new predictive methods [5]. The prediction of protein–protein interactions is vital for accurately positioning proteins within signalling pathways and networks. The importance of the development of reliable predictive tools continues to increase with the explosion of genomic and proteomic data [6]. Over the past few decades, the critical assessment of techniques for protein structure prediction (CASP) [7], the critical assessment of prediction of interactions (CAPRI) [8] and the continuous automated model evaluation (CAMEO) [9] prediction experiments have acted as a catalysis spurring the development of new quaternary structure modelling techniques. These experiments take the form of competition, and the results allow researchers to objectively assess the quality of different methods for prediction of protein structures, functions and interactions. In recent years the CASP experiment has included the assessment of quaternary structures, where predictors are encouraged to submit the oligomeric assemblies of the protein targets. Furthermore, the recent addition of the Data Assisted category in CASP aims to evaluate how much the accuracy of 3D models can be improved by the addition of sparse data from NMR, crosslinking, and SAXS experiments.

## 2. Experimental Methods for Determining Protein–Protein Interactions (PPIs)

Various rapid bioinformatics tools are available for the inherently complex process of PPI prediction, yet experimental methods are still considered more reliable due to the current accuracy limitations of computational methodologies. However, determining the full atom coordinates for a protein complex or quaternary structure, for example using X-ray crystallography, is very costly and time-consuming and the crystallisation process itself can be hit and miss. Alternative experimental techniques for PPI detection are more reliable, but they do not provide detailed atomic information of quaternary structures. Therefore, there is a growing need for techniques that aim to fuse sparse experimental data with predictive data to aid with the modelling of complexes.

**Figure 1:**
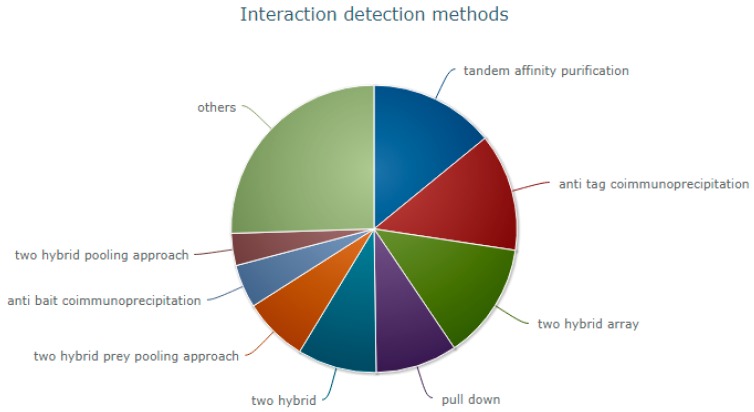
Interaction detection methods.

As per the statistics from IntAct [10], an open source database system for molecular interaction data, 523,010 interactions (either curated from the literature or direct data depositions) have been submitted until 3 October 2017. The experimental interaction detection methods can be broadly classified into: (i) biochemical; (ii) biophysical; (iii) genetic interference; (iv) imaging technique; (v) phenotype based detection assays; (vi) post-transcriptional interference; and (vii) protein complementation assays. The most common techniques as per IntAct are illustrated in Figure 1. In this section, we focus on advantages and limitations of each method.

Tandem affinity purification (TAP) is a biochemical PPI detection method, which accounts for 14.1% of the experimental methods as per IntAct [10]. TAP is a proteomic high throughput technique, which was initially developed in yeast, but can be adapted to a variety of organisms. Wide applicability and simplicity make TAP a useful method for protein purification [11]. This method involves fusion of TAP tag, either N- or C-terminally to the target protein of interest, which is then encoded into the organism, where they form physiological complexes with other proteins. SDS-PAGE and mass spectrometry is then used to examine and identify proteins extracted with the tagged bait [12]. In this highly sensitive and selective method, transient or weak interactions may be lost during the series of purification stages and requirement of a large number of samples is also a disadvantage as it is difficult to purify and identify low abundance binding partners [13]. TAP is used to detect multiple PPIs at one time, but errors can be expected due to the interference of tags in proteins, which can be overcome by subsequent characterization [14].

If antibodies against the target protein are available, Co-immunoprecipitation (Co-IP) is considered to be a simple technique to study PPIs in vivo [15]. In this method, a protein-specific antibody is incubated with a protein mixture, which forms an immune complex with the target protein. Target proteins may interact with other proteins to form a protein complex, which is then immunoprecipitated using immobilized Protein A or Protein G [16]. As protein complexes can be part of large complexes, the co-immunoprecipitating proteins may not interact directly. Due to the lower concentration of antigen, precipitation is not as intensive as other methods [17]. Another disadvantage of this biochemical method is that during the elution of precipitated antigen, it releases antibody which contaminates the antigen [15,16].

Yeast two-hybrid is a high throughput PPI detection method, which accounts for 9.2% of the PPI detection methods submitted in IntAct [10]. In this genetic interference method, which was described initially by Field and Song in 1989, functional transcription factors formed by the interaction between bait and prey protein induces the specific reporter gene expression, allowing the detection of such interactions [18]. Despite being simple to set up, low cost and useful in detecting transient and weak interactions [19], the estimated the high rate of false positives (as high as 50%) suggests any detected interactions must be further verified by other methods [20]. Also, proteins, which are less likely to be present in the nucleus, are excluded, as proteins must be localised to the nucleus for this method. Furthermore, proteins that are not in their natural physiological environment may not fold correctly to interact [21].

The pull-down method, which involves the use of affinity purification, is similar to CoIP in methodology, i.e., uses affinity ligand to capture interacting proteins. This method uses “bait”, purified and tagged protein in place of immobilised antibodies in CoIP [22]. Interactions mediated between the immobilized bait protein and a target protein by the negatively charged nucleic acid, which stick to the basic surface on the protein, may generate a false positive result in protein–protein interaction assays [23].

In protein chip technology, expressed purified and screened proteins are printed onto a chip using a microarrayer as discrete spots. A solution containing labelled proteins is incubated with the chip, which is then washed and the position of the labels indicates the interaction between proteins (protein on the chip and protein from the solution) [24]. High signal to noise ratio, high sensitivity and the relatively small quantity of sample requirement makes protein chip technology preferable over other techniques, but the proteins attached to the chip can disrupt protein interactions [25,26]. Even though, current advances in the manufacture of protein chip technology use glass microscope slides and other materials that allow the protein to attach on their surface at high density, the technological challenges in this field still remain [24].

Even though X-ray crystallography is the most popular method of protein structure determination, NMR spectroscopy has played a vital role where protein complexes have disorders and when challenging to obtain a crystal. NMR is handy in weak detection PPIs (K_d_ > ~100 μM) and hence is a method preferred to study PPI in excellent detail [27]. As this biophysical technique gives atomic level information in the solution state, this method is of particular interest for PPI analysis by researchers [28].

Each experimental technique has its strengths. However, most of these techniques require expensive and extensive instrumentation and specific knowledge to analyze the results. Hence the field is developing and new and improved methods frequently emerging. An overview of the current experimental methods available is shown in Table 1. 

### Sparse Experimental Data on Prediction of Protein Interactions and Modelling of Their Complexes

SAXS can provide a wealth of structural information on biomolecules in solution [70]. Advanced usage of SAXS can provide a unique insight into biomolecular behaviour that can only be observed in solution, like transient protein–protein interactions [71]. As SAXS data miss the relevant atomic details, the low-resolution information can be combined with theoretical methods which give the energetic description and atomic details of the interactions [72].

Tang et al. used a method by combining sparse NMR data with evolutionary couplings (ECs), which involves simultaneous analysis of ECs, derived from multiple sequence alignments (MSAs), with NMR chemical shift, nuclear Overhauser effect (NOE) and residual dipolar coupling (RDC) data. The results with this EC-NMR method were said to be more accurate and provided complete structural information compared to the ones obtained with sparse NMR data or ECs alone [73].

In 2007, Latek, Ekonomiuk, and Kolinski combined de novo modelling with limited experimental data and demonstrated that limited experimental data, such as chemical shifts, are sufficient for three-dimensional structure determination if the data are accurate. It was also suggested that weak quality chemical shift data can be improved by the application of additional sparse experimental data [74].

Recently, an integrative modelling approach was used to determine the 3D model of the entire Mediator complex. Robinson et al. combined data from X-ray crystallography, homology modelling, and cryo-electron microscopy with the results from chemical cross-linking and mass spectrometry to produce the model. All Mediator subunits, subunit interfaces and some secondary structural elements were revealed in the elucidated model [75].

Cross-linking is a biochemical PPI detection method, which is suitable for detecting weak interactions. As the reagent used in this technique detects interactions which are not in direct contact, the interactions detected with this method need to be assessed independently. Combined with mass spectrometry, chemical cross-linking can be used to study PPIs. In purified protein complexes they are used to study spatial relationships. Both transient and stable interactions can be studied using the information from the controlled arrays produced using new classes of photo-activated reagents [76,77].

The requirement of only a small amount of sample and the lack of a requirement for crystallisation has made cryo-electron microscopy (cryo-EM) a method preferable over other techniques [78,79]. Statistics from the Electron Microscopy Data Bank, a public repository for electron microscopy density maps of macromolecular complexes and subcellular structures indicates that cryo-EM is becoming a favourite structural biology technique to study challenging biological systems [80]. The high cost of EM equipment, the requirement of expensive maintenance and enormous computational resources are the current challenges of cryo-EM [78]. However, cryo-EM is suggested as an ideal platform for the integration and modelling of data from other experimental methods for structural biology [81]. Hong-Wei Wang and Jia-Wei Wang explain the technique as a complementary method to X-ray crystallography. They describe how intermediate resolution maps from cryo-EM can assist in X-ray crystallography structure determination of macromolecular complexes [82]. Ning Gao and co-workers used a hybrid method combining structural information, chemical cross-linking proximity mapping followed by mass spectrometry (CX-MS) and cryo-EM for the modelling of five assembly factors in the ITS2 region of the pre-60S ribosome [83]. Residue-level details of PPI of dengue virus coat proteins was predicted using Cα atom positions from low-resolution cryo-EM structures [84,85].

Experimental methods have proven invaluable to further our understanding of PPIs. However, each method has its disadvantages. Also, the relatively high cost, time and expertise requirements of in vitro techniques suggest that in silico methods will need to play an increasingly important role for understanding protein–protein interactions at the atomic level.

## 3. In Silico Methods for Modelling Protein–Protein Complexes

There are two primary categories of methods for modelling protein–protein complexes: (1) homology or template-based modelling and (2) ab initio docking, or template-free modelling. Template-based modelling relies on the existence of appropriate templates in protein databases, and docking approaches can be broadly split into rigid body and flexible solutions. Docking guided by restraints obtained from experimental procedures could be considered a third category and is often referred to as “hybrid modelling”. However, the computational pipelines used in any integrative approach would usually comprise either template modelling or docking, or both. For protein structure prediction, ab initio methods have gained importance because the number of quaternary structural models available in the Protein Data Bank (PDB) [86] limits the well-established traditional method of template-based modelling, which is successfully routinely used for tertiary structure prediction. This is true despite the fact that the PDB is growing at an increasing rate due recombinant DNA methods allowing large amounts of target biological macromolecules to be expressed for analysis [2]. 

The increase in the rate of sequencing of biological macromolecules means that ab initio methods for determining structures and interactions of proteins are becoming more relevant. Protein docking is the computational determination of protein complex structure from individual protein structures. When using docking software, there are two classes of problems: bound docking, where the complex structure is known and the receptor and ligand are pulled apart then reassembled, and unbound docking, where individually determined proteins structures are used. Docking is computationally expensive, and the accuracy can decrease rapidly when the protein chains undergo significant conformational changes upon binding. A brief description of the variety of different methods utilized in homology modelling and docking follows, the methods have been chosen to be representative of some common successful methods as well as some less common approaches.

### 3.1. Computational Methods for Protein–Protein Interaction Modelling

RosettaDock is a soft body type of protein interaction prediction software and considers backbone or side-chain flexibility, which is advantageous regarding obtaining structures of protein complexes with high-resolution accuracy. The protein docking server predicts the structure of protein complexes given the compositions of the individual components and a relative binding orientation. The server identifies low-energy conformations of a protein–protein interaction near a given starting configuration by optimising rigid-body orientation and side-chain structures [87]. RosettaDock has made a substantial effort in developing flexible-backbone docking approaches in CAPRI rounds 6–12. However, the rigid-body ZDOCK [88] approach was more successful for a number of the targets. ZDOCK and other approaches employing softer potentials are less sensitive to backbone conformational changes. 

ZDOCK, which is presently one of the most common rigid-body docking engines, uses a scoring function that includes shape complementarity, electrostatics and a heuristic potential called atomic contact energy [88,89,90]. The combination of specific backbone optimization plus a strict potential should be optimal in the high sampling limit where the correct conformation is sure to be encountered, but with insufficient sampling, the results can be inferior to fixed-backbone sampling with a soft potential [91]. A recent development of RosettaDock is the use of SAXS data as a constraint step in selecting predicted complexes by using the SAXS data to reject complexes that violate shape constraints imposed by the SAXS data [92] thereby increasing the accuracy of final predictions.

Rigid body docking as a method produces a large number of docked conformations with favorable surface complementarity usually followed by ranking using energy scoring. The fast Fourier transform (FFT) algorithm uses a correlation approach [93] and systematically explores the space of possible docked conformations using electrostatic interactions or both electrostatic and solvation terms, but the potential is restricted to a correlation function form. FFT approaches are very common and include GRAMM-X which extends the original GRAMM FFT method by employing smoothed potentials, refinement stage, and knowledge-based scoring [94]. The Hex Protein Docking Server (HexServer) is an FFT based protein-docking server that utilizes graphics processing unit (GPU) acceleration. By using multiple GPUs, a typical docking run takes approximately 15 s, which is up to two orders of magnitude faster than conventional FFT based docking approaches using comparable resolution and scoring functions [95] such as GRAMM-X, FRODOCK and MEGADOCK.

MEGADOCK [96] uses a Katchalski–Katzir algorithm [93] and searches probable docking structures in a grid-based 3D space using FFT. MEGADOCK employs a scoring function in which only shape complementarity and electrostatics are considered and thus makes the calculations 8.8 times faster than ZDOCK. The method is set up to perform massive numbers of calculations that are run on parallel computing systems, and along with GPU.proton.DOCK and HexServer demonstrates the increase in search speed when using hardware acceleration.

FRODOCK projects the interaction terms of a potential protein complex into 3D grid-based potentials using spherical harmonics approximations to accelerate the search; this is itself an extension of the FFT alogrithm. The binding energy of the complex as it is formed is approximated as a correlation function composed of van der Waals, electrostatics and desolvation energy terms [97] and this is used for the final ranking in FRODOCK. In FRODOCK 2.0 a new complementary coarse-grained knowledge-based protein-docking potential was introduced [98] and this two-step contact potential was incorporated because of the excellent trade-off between accuracy and low computational cost [99].

M-ZDOCK was developed for predicting the structure of cyclically symmetric multimers based on the construction of an unbound (or partially bound) monomer. Using a grid-based FFT approach purely symmetrical multimers are generated and searched for the highest quality structure rather than creating predicted structures with ZDOCK [88,89] and filtering for adjacent symmetrical structures. Fewer false positives are considered in the search, therefore, reducing the number of overlooked high-quality structures and the amount of computing power required is reduced by searching four instead of six degrees of freedom. Testing known multimer complexes from the PDB, M-ZDOCK was able to find at least one high-quality structure, whereas only two of the four test cases did when using ZDOCK and a filter for symmetry, and the running times are 30–40% faster for M-ZDOCK [100]. M-ZDOCK demonstrates how limiting focus can improve results, which calls for an integrative approach using many methods to cover different eventualities.

The ClusPro [101] docking algorithm evaluates multiple presumed complexes, retaining a pre-set number with encouraging surface complementarities, next a filtering method is applied to this set of structures, selecting those with good electrostatic and DE free energies for further clustering [102]. ClusPro-DC is a recent development of the ClusPro method which discriminates between crystallographic and biological dimers by docking the two subunits to sample the interaction energy landscape exhaustively [101,103]. The ClusPro server also utilizes SAXS data as constraints for selecting possible predicted structures [104] and has demonstrated an improvement in accuracy whilst doing so.

PatchDock [105] and LZerD [106] both utilize non FFT based methods for PPI prediction and could elucidate relevant predicted complexes that FFT based solutions may be missing due to the vagaries of mathematics. The PatchDock algorithm was inspired by object recognition and image segmentation techniques, where two molecules have their surfaces divided into patches based on the surface shape [105]. The surface of the proteins is calculated, a segmentation algorithm for detection of geometric patches (concave, convex and flat surface pieces) is applied and the patches are filtered, so that only patches with residues involved in binding are retained. A surface patch matching procedure applies geometric hashing and pose clustering matching techniques to match the patches previously detected, concave patches are paired with convex and flat patches with any patches. The predicted complexes of the previous stage are examined; all complexes with unacceptable clashes between receptor atoms and ligand atoms are discarded. Finally, the remaining candidates are ranked according to a geometric shape complementarity score [107]. 

The LZerD [106] software suite provides both pairwise dockings with LZerD and asymmetric multimeric docking with Multi-LZerD. LZerD has demonstrated improved performance since its introduction to the CAPRI experiment due to its continued integration of template based modelling, docking and scoring functions [108,109]. Multimeric docking programs have limited themselves to symmetrical complexes, which makes Multi-LZerD particularly useful as it provides complex asymmetric predictions. This is achieved using pairwise docking predictions from LZerD utilising the 3DZD a rotational invariant mathematical surface representation of proteins [106]. These are then combined using a genetic algorithm and several scoring methods are used, including clashing of atoms determined by atoms being with 3.0 Å of each other in each subunit. Furthermore, a physics-based scoring system that incorporates multiple scoring terms, where repulsive and attractive parts of the term are considered separately; an electrostatics term, which considers repulsive/attractive and short-range/long-range contributions individually; a hydrogen and disulphide bond term; two solvation terms; and a knowledge-based atom contact term [109].

Table 2 summarises the various bioinformatics methods for PPI modelling and how to access them. 

### 3.2. Quality Assessment of Quaternary Structure Predictions

Quality assessment is vital to structure prediction as it allows us to reduce a potentially considerable number of predicted complexes to a smaller subset for further ranking or examination. Energy based scoring of protein complexes is often carried out by docking programs to filter predictions and takes into account several binding terms: van der Waals energy and shape complementarity; desolvation energy and hydrophobicity; electrostatic interaction energy; translational, rotational and vibrational free energy changes. The assumption made by energy scoring is that proteins will find the lowest energy configuration when forming complexes. However, this is not always true, as some complexes observed by experimental methods have not taken up the lowest possible energy pose. Furthermore, these energy functions provide relative accuracy estimates, with only moderate power in adequately ranking models. Further, when one tries to compare models obtained from different methods, their associated energy scores are often not directly comparable. Therefore, accurate quality estimation methods that are independent of energy scoring are essential for protein structure prediction tools to fulfil their potential as useful techniques for biologists [90].

Consensus clustering can be used to determine the quality of predicted protein complexes. Docking predictions for pairs or groups of protein chains are clustered based on a scoring system that compares the similarity of the predictions using either root mean square deviation (RMSD) of the distance between the atoms of the complex in angstroms or the TM-score [112] of determining the similarity between multimeric protein complexes. Two complexes are considered to be neighbors if they are closer than a threshold angstrom (Å) value and are then placed into a cluster. Once the clusters are created, a prediction is selected out of each cluster as a representative prediction, using shape or physics-based scoring methods, and then the other predictions in the clusters are deleted. Through repeated passes of this process, the total number of predicted poses can be reduced to a more manageable number and processed manually to determine the highest quality predicted pose.

Although many different forms of tertiary model evaluation exist; such as those implemented in ModFOLD6 [113], ProQ3 [114,115] and others [116], recent progression onto quaternary protein modelling means that a reliable generic quaternary structure model quality assessment method has yet to be developed. The ZDOCK method uses an energy-based ranking system that is available separately from the ZDOCK prediction software [90]. ZRANK has also been integrated into MEGADOCK for ranking of predicted structures [96,117]. Similarly, upon evaluation of protein modelling in CASP11 [118], final quaternary model evaluation was recognized as an important but neglected area in need of significant development. Towards the end of the CASP12 experimental cycle, a new category for assessing the quality of the interface in protein–protein interactions was proposed. A preliminary scoring system, ModFOLD IA, for evaluating MultiFOLD models was trialed by the McGuffin group, following the submission of their top submitted complexes for CASP12. The ModFOLD IA method generates interface accuracy scores (IAs) for individual interface residues in MultiFOLD quaternary models. This is achieved first by identifying interface residues (heavy atoms within <5 Å of each other on different protein chains), followed by calculation of the minimum possible distance between residues in each interface pair (Dmin), a comparison of the mean Dmin between each interface pair across alternative models for each protein target (MeanDmin), and finally the generation of an IA score based on the difference between Dmin and MeanDmin per interface residue within each alternative protein model. This simple score aimed to provide a basis for interface residue scoring across generic alternative oligomer models by comparison of the interfaces in individual models to that of the mean interface value for a given target.

## 4. CASP, CAPRI and CAMEO: Driving the Development of in Silico Quaternary Structure Prediction Methods and Their Fusion with In Vitro Data

The development of methods for the prediction of protein-ligand binding sites and function prediction has been supported in recent years as a direct result of community-wide prediction experiments, such as CASP [7], CAPRI [8] and CAMEO [9].

### 4.1. CASP

CASP1, the first large-scale experiment to assess protein structure prediction methods, was held in 1994. It consisted of three parts: the collection of targets for prediction from the experimental community, the group of forecasts from the modelling community, and the assessment and discussion of the results. Information was solicited from X-ray crystallographers and NMR spectroscopists on structures about to be solved. Protein–protein interaction prediction was not added to CASP until CASP11 in 2014, however, in the years since, the area has proliferated and was a much more significant part of CASP12 in 2016. CASP has a large number of target sequences for prediction and runs over several months, culminating in a conference to review the experiment. 

**Figure 2:**
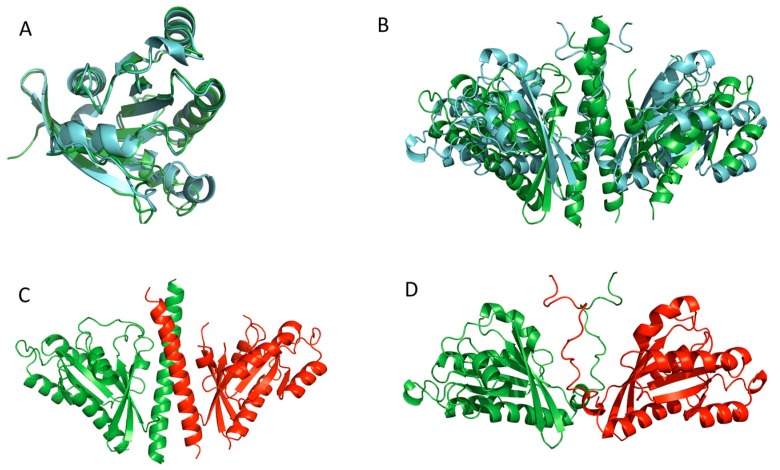
The challenge of making the transition from tertiary to quaternary structure prediction—a homodimer prediction. Even with a high quality starting tertiary structure other factors, such as disorder-order transitions, may come into play. (**A**) Superposition of Truncated Predicted and Truncated Observed Monomers of T0798 target Sequence with the observed monomer colored blue and the predicted monomer colored green; (**B**) Superposition of Predicted and Observed Dimer of T0798 target Sequence with the observed dimer coloured green and the predicted dimer colored blue, aligned with PyMOL; (**C**) Observed Dimer of T0798 Target Sequence with each of the two monomers coloured green and red respectively; (**D**) Predicted Dimer of T0798 Target Sequence with each of the two monomers coloured green and red respectively.

In Figure 2 computationally predicted and experimentally observed structures for a target protein from the CASP11 preliminary round are shown, highlighting some of the challenges of making the step from tertiary to quaternary structure prediction. The experimentally observed structure was provided by CASP, the target multimer is a dimer of protein Rab11b observed by X-ray spectroscopy and 198 residues in length, and the predicted structures were generated by the McGuffin group using a simple homology modelling procedure. Figure 2A shows the superposition generated by TM-align [112] of the observed and predicted monomers, which have been truncated by removing the disordered region from the predicted structure and the alpha helix from the observed structure. This superposition has a TM-score of 0.93365, which demonstrates the high quality of the initial tertiary structure prediction for this target sequence. The superposition of the complex shown in Figure 2B generated a TM-score of 0.42037 when aligned using MM-align [119]. In Figure 2D the predicted structure shows a disordered region that was predicted to form the interaction site for the dimer. In Figure 2C, the observed structure shows that the disordered regions formed a stable alpha helix upon dimerization. The dimer superposition in Figure 2B has a much lower TM-score than the truncated monomer superposition due to the disordered region becoming ordered and an orientation change about the disordered region, which has not allowed the predicted and observed monomers to align as well as they can be aligned when truncated.

**Figure 3:**
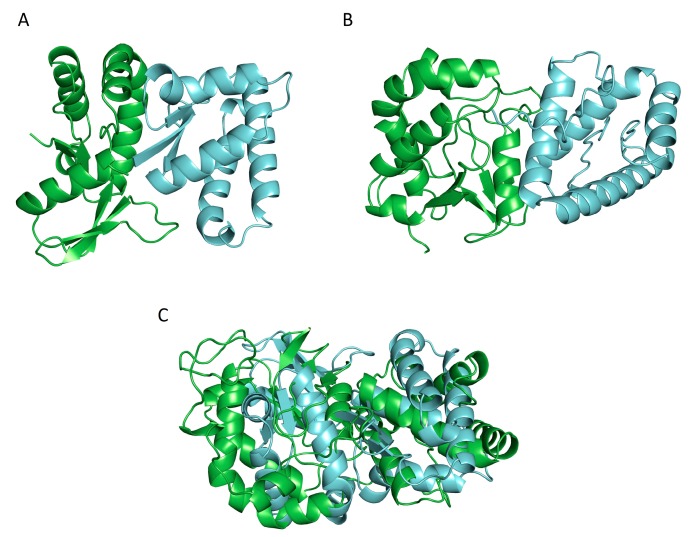
The challenge of making the transition from tertiary to quaternary structure prediction—a hetero dimer prediction. The success of modelling a complex is reliant on the quality of the starting tertiary structure model. (**A**) Observed structure of T0868/T0869 dimer; (**B**) Predicted structure of T0868/T0869 dimer from poor quality initial tertiary structures; (**C**) Superposition of observed and predicted T0868/T0869 dimer with the observed dimer coloured green and the predicted dimer colored blue, aligned with PyMOL.

In the CASP 12 experiment the McGuffin group predicted tertiary structures using IntFOLD [120,121,122,123] and other server models, then subsequently the highest scoring submitted model was further refined using the ReFOLD [124] procedure. The ReFOLD refinement pipeline consists of three main protocols. The first and second protocol used i3Drefine [125] and NAMD [126] for structure refinement of the starting model. The generated refined models were then assessed and ranked using the new and improved ModFOLD6 [113,127,128]. From this, only top 5 refined models were selected and submitted for the CASP12 experiment, and the top model was used for MultiFOLD analysis. MultiFOLD is an as yet unpublished meta-server method for determining protein quaternary structure that utilises the LZerD, MEGADOCK, FRODOCK, PatchDock and ZDOCK for dimeric complexes (two protein subunits) and M-ZDOCK and Multi-LZerD for multimeric complexes (three or more protein subunits). The predicted quaternary structures were then ranked for submission using a variety of methods including each programs internal ranking procedures, other ranking software and manual intervention using information provided by CASP concerning the origin of the protein. In Figure 3 computationally predicted and experimentally observed structures for a target protein (T0868/T0869 heterodimer) from the CASP 12 preliminary round are shown. The experimentally observed structure, Figure 3A, was provided by CASP and the predicted structure, Figure 3B was generated by the McGuffin group using the MultiFOLD method. A superposition of the observed and predicted structures for the dimer is shown in Figure 3C and has a TM-score of 0.33088 indicating a low similarity between the two structures.

The ultimate goal of structure prediction is to provide insights into biological functions. However, it is difficult to quantify and benchmark the utility of protein structure prediction for functional inference [129]. The biological function of a protein may have several different meanings; it can include catalyzing chemical reactions, structural support. Most transporting materials across the cell, receiving and sending chemical signals, or responding to stimuli and providing of these functions are realised by interacting with other proteins or small molecules. Therefore, interfaces between proteins, or interfaces between a protein and small molecules are critical to understanding function. Official CASP structural assessments include global and local metrics that evaluate the atomic level similarity of the structural features of proteins [130,131,132]. The root mean square deviation (RMSD) was the first metric used in the CASP evaluations, and it is still reported in the automatic evaluation system. The global distance test (GDT) score is useful for the automatic evaluation of predictions as it reflects the absolute and relative accuracy of models for a wide range of target difficulty. In addition to GDT, several other similarity measures are used. Structural quality often tracks with functional quality, but the details of this correlation need to be further explored. 

During CASP11, it was seen that the model accuracy was improved when using NMR simulated sparse data. Dramatic improvement was seen in GDT_TS score (Global Distance Test used to quantify prediction performance) for all data assisted models, where GDT_TS was better by >17 GDT_TS units on average compared with the CNS (crystallography and NMR system) results (those models unassisted by data) [133].

### 4.2. CAMEO

CAMEO continuously applies quality assessment criteria established by the protein structure prediction community. Since the accuracy requirements for different scientific applications vary, there is no “one fits all” score. CAMEO, therefore, offers a variety of scores—assessing different aspects of a prediction (coverage, historical accuracy and completeness) to reflect these requirements [9]. CAMEO operates on a continuous automated basis and is therefore only suitable for server-based predictive methods. The Contact Prediction (CP) section of the CAMEO experiment has been running for 56 weeks with four groups registered as predictors, and 68 target sequences have been tested.

### 4.3. CAPRI

CAPRI is a blind prediction experiment, and its targets are unpublished crystal or NMR structures and are submitted to CAPRI by their authors in a confidential process to maintain the validity of the experiment. Contributor predictor groups are given the atomic coordinates of two proteins that are known to have relevant biological interactions. The target interactions are modelled with the help of the coordinates and other publicly available data and then submit sets of ten models for assessment. Also, the predictors are invited to upload more massive sets that are communicated to scorer groups who evaluate and rank them and make a separate ten-model submission. After the prediction round is finalised, the CAPRI assessors equate the submissions to the experimental structure and evaluate the models on criteria that depend on the geometry and biological importance of the predicted interactions [8]. CAPRI has four prediction rounds each year with only a few targets per round but is growing with each round.

## 5. Conclusions

A large number of high throughput experimental techniques and computational programmes are now available for both PPI detection and modelling quaternary structures. Integrating this information together with sparse experimental data, or hybrid modelling, will help biologists to elucidate the complex networks of PPIs, which are intrinsic to every cellular process. A plethora of methods are being explored for in silico quaternary structure prediction and the refinement and quality assessment of such predictions. In docking, methods based on the FFT algorithms are currently prevalent although the field is developing alternative methods. Energy scoring is common to the majority of methods as the main ranking criteria and clustering techniques are also sometimes utilized. However, there is a clear need for the development of generic quality assessment methods for evaluating predicted quaternary structures, if models are to be more seriously adopted by the wider biology community. The combination of predictive and experimental methods and the continued focus of the CASP, CAPRI and CAMEO on quaternary structures, will be essential for driving our continued progress.

## Figures and Tables

**Table 1:** Overview of experimental methods for PPI detection and their categories.

Interaction Detection Method	Current Available Techniques	Reference
**Biochemical**	Affinity technology	[29]
	Aggregation assay	[30]
	Chromatography technology	[29]
	Cosedimentation	[29]
	Cross-linking study	[29]
	Electrophoretic mobility-based method	[31]
	Enzymatic study	[29]
	Footprinting	[29]
	Nucleotide exchange assay	[32]
	Polymerization	[33]
	Probe interaction assay	[29]
**Biophysical**	Biosensor	[34]
	Circular dichroism	[35]
	Mass spectrometry	[29]
	Differential scanning calorimetry	[36]
	Electron diffraction	[37]
	Electron resonance	[29]
	Enzyme-mediated activation of radical sources	[38]
	Equilibrium dialysis	[39]
	Filter trap assay	[40]
	Fluorescence technology	[30]
	Infrared spectroscopy	[41]
	Intermolecular force	[42]
	Isothermal titration calorimetry	[43]
	Light scattering	[44]
	Luminescence technology	[45]
	Microscale thermophoresis	[46]
	Molecular sieving	[17]
	Neutron diffraction	[47]
	Neutron fibre diffraction	[48]
	Nuclear magnetic resonance	[49]
	Rheology measurement	[50]
	Scintillation proximity assay	[51]
	Small angle neutron scattering	[35]
	Thermal shift binding	[52]
	Ultraviolet- visible spectroscopy	[53]
	X-ray crystallography	[29]
**Genetic interference**	Chemical RNA modification plus base	[54]
	Random spore analysis	[29]
	Synthetic genetic analysis	[29]
**Imaging techniques**	Atomic force microscopy	[55]
	Confocal microscopy	[29]
	Electron microscopy	[56]
	Fluorescence microscopy	[29]
	Light microscopy	[29]
	Super-resolution microscopy	[29]
	X-ray tomography	[29]
**Phenotype-based detection assay**	Nuclear translocation assay	[57]
**Post-transcriptional interference**	Antisense oligonucleotides	[29]
	Antisense RNA	[58]
	RNA interference	[59]
**Protein complementation assays**	Adenylate cyclase complementation	[60]
	β-galactosidase complementation	[61]
	β lactamase complementation	[62]
	Bimolecular fluorescence complementation	[63]
	Dihydrofolate reductase reconstruction	[64]
	Mammalian protein–protein interaction trap	[65]
	Protein kinase A complementation	[66]
	Reverse ras recruitment system	[67]
	Split luciferase complementation	[68]
	Tox-R dimerization assay	[69]
	Transcriptional complementation assay	[29]

**Table 2:** Overview of bioinformatics methods for modelling PPIs.

Name	Method	URL	Reference
RosettaDock	RosettaDock is a Monte Carlo (MC) based multi-scale docking algorithm.	https://www.rosettacommons.org/	[87]
ZDOCK	FFT used to perform a 3D search of the spatial degrees of freedom between two molecules., utilizes a pairwise statistical potential in the scoring function.	http://zdock.umassmed.edu/	[110]
GRAMM-X	The best surface match between molecules is determined by correlation technique using FFT, uses a smoothed Lennard-Jones potential on a fine grid during the global search FFT stage.	http://vakser.compbio.ku.edu/resources/gramm/	[94]
HexServer	Uses a closed-form 6D spherical polar FFT correlation expression from which arbitrary multi-dimensional multi-property multi-resolution FFT correlations may be generated.	http://hexserver.loria.fr/	[95]
MEGADOCK	MEGADOCK uses a Katchalski-Katzir algorithm and searches probable docking structures in a grid-based 3D space using FFT. MEGADOCK employs a scoring function in which only shape complementarity and electrostatics are considered. The method is set up to perform massive numbers of calculations that are run on parallel computing systems.	http://www.bi.cs.titech.ac.jp/megadock/	[96]
FRODOCK	FRODOCK projects the interaction terms of a potential protein complex into 3D grid-based potentials using spherical harmonics approximations to accelerate the search; this is itself an extension of the FFT alogrithm.	http://frodock.chaconlab.org/	[97]
M-ZDOCK	A grid-based FFT approach generates symmetrical multimers which are searched for the highest quality structure rather than creating predicted structures with ZDOCK and filtering for adjacent symmetrical structures.	http://zdock.umassmed.edu/m-zdock/	[100]
ClusPro	The ClusPro docking algorithm evaluates multiple presumed complexes, retaining a pre-set number with encouraging surface complementarities, next a filtering method is applied to this set of structures, selecting those with good electrostatic and DE free energies for further clustering.	https://cluspro.bu.edu/home.php	[101]
Patchdock	Two molecules have their surfaces divided into patches based on the surface shape. The surface of the proteins is calculated, a segmentation algorithm for detection of geometric patches is applied and the patches are filtered, so that only patches with residues involved in binding are retained. A surface patch matching procedure applies geometric hashing and pose clustering matching techniques to match the patches previously detected.	https://bioinfo3d.cs.tau.ac.il/PatchDock/	[105]
LZerD	Uses the 3DZD a rotational invariant mathematical surface representation of proteins to generate predictions.	http://www.kiharalab.org/proteindocking/lzerd.php	[111]
Multi-LZerD	Uses pairwise docking predictions from LZerD, these are then combined using a genetic algorithm and several scoring methods are used.	http://kiharalab.org/proteindocking/multilzerd.php	[106]

## Abbreviations

NMR	Nuclear magnetic resonance
SAXS	Small-angle X-ray scattering
PPI	Protein–protein interaction
TAP	Tandem affinity purification
Co-IP	Co-immunoprecipitation
CD	Circular Dichroism
ECs	Evolutionary couplings
GPU	Graphics processing unit
NOE	Nuclear Overhauser effect
RDC	Residual dipolar coupling
Cryo-EM	Cryo-electron microscopy
PDB	Protein Data Bank
RMSD	Root mean square deviation
CASP	Critical Assessment of techniques for Structure Prediction
CAPRI	Critical assessment of prediction of interactions
CAMEO	Continuous automated model evaluation
LDDT	Local distance difference test on all atoms
3DZD	3D Zernike descriptor
FFT	Fast Fourier transform
